# Identification of an Allosteric Binding Site on Human Lysosomal Alpha-Galactosidase Opens the Way to New Pharmacological Chaperones for Fabry Disease

**DOI:** 10.1371/journal.pone.0165463

**Published:** 2016-10-27

**Authors:** Valentina Citro, Jorge Peña-García, Helena den-Haan, Horacio Pérez-Sánchez, Rosita Del Prete, Ludovica Liguori, Chiara Cimmaruta, Jan Lukas, Maria Vittoria Cubellis, Giuseppina Andreotti

**Affiliations:** 1 Dipartimento di Biologia, Università Federico II, Napoli, 80126, Italy; 2 Bioinformatics and High Performance Computing Research Group (BIO-HPC), Computer Engineering Department, Universidad Católica San Antonio de Murcia (UCAM), Spain; 3 Istituto di Chimica Biomolecolare–CNR, Pozzuoli, 80078, Italy; 4 Albrecht-Kossel-Institute for Neuroregeneration, Medical University Rostock, Rostock, Germany; Louisiana State University Health Sciences Center, UNITED STATES

## Abstract

Personalized therapies are required for Fabry disease due to its large phenotypic spectrum and numerous different genotypes. In principle, missense mutations that do not affect the active site could be rescued with pharmacological chaperones. At present pharmacological chaperones for Fabry disease bind the active site and couple a stabilizing effect, which is required, to an inhibitory effect, which is deleterious. By *in silico* docking we identified an allosteric hot-spot for ligand binding where a drug-like compound, 2,6-dithiopurine, binds preferentially. 2,6-dithiopurine stabilizes lysosomal alpha-galactosidase *in vitro* and rescues a mutant that is not responsive to a mono-therapy with previously described pharmacological chaperones, 1-deoxygalactonojirimycin and galactose in a cell based assay.

## Introduction

Fabry disease (FD) is a rare pathology, but accounts for 8.8% of the patients affected by inherited disorders of metabolism [1]. It is caused by mutations in the gene *GLA*, which is located on the X chromosome and encodes lysosomal alpha-galactosidase (AGAL) [2]. Enzyme replacement (ERT) is the only approved specific therapy for FD; it is well tolerated and safe [3]. The large phenotypic and genotypic spectrum of the disease poses several problems and the cost-effectiveness of ERT is still debated, in particular for patients who have residual enzyme activity [4]. In FD, about 40% of all missense mutations are associated with a biochemically mildly damaged enzyme [5]. A therapy with sub-inhibitory concentrations of 1-deoxygalactonojirimycin (DGJ) was first proposed by Suzuki and co-workers [6] and then tested in cells [7–13], in mouse models of FD [14] [15] and in clinical trials [16] [17]. Such therapy is suitable only for patients carrying specific mutations (40%-60% of missense mutants tested in cell based assays [11, 12]) and requires fine-tuning of the dosage regimen because DGJ is an inhibitor of AGAL. The approach with small molecules for FD is promising, but DGJ is not yet the ideal drug. Molecules that either substitute DGJ or act in synergy with it to enable reduced dosage of DGJ should be sought, in particular for mutants that do not respond to a monotherapy with DGJ. Analogues of DGJ have been developed [18] [19]. A new family of arylthioureas showed a better balance between the stabilizing effect, which is required, and the inhibitory effect, which is detrimental [20]. However, these molecules bind the active site, as demonstrated by x-ray crystallography, and enhance enzymatic activity only at micromolar concentration, not dissimilarly from DGJ, when administered to eukaryotic cells expressing AGAL mutants [20].

Ambroxol, a mucolytic agent used in the treatment of respiratory diseases, proved to be useful in association with DGJ to rescue some AGAL mutants [21]. Its mechanism of action is not clear since ambroxol is not specific for AGAL, but it is effective also on lysosomal beta-glucocerebrosidase [22] and alpha-glucosidase mutants [21].

Discovery of chaperones directed against the active site is facilitated by focusing on molecules with structural similarity to galactose, a natural product of AGAL. Conversely, the discovery allosteric ligands, i.e. ligands that bind AGAL at sites outside the active site, is complicated by the fact that they may not chemically resemble any known substrates or products. We performed *in silico* molecular docking (virtual screening) of over ten thousand low molecular weight structurally diverse compounds previously filtered from ZINC database [23], by looking for molecules that bind preferentially at a site different from the active site. Among the top ranking hits we found one molecule, 2,6-dithiopurine (DTP). This molecule was chosen for further studies since it has already passed some safety tests, is a known chemiopreventive agent [24] [25] and it is safe when administered to mice [26]. DTP is able to stabilize AGAL against thermal and urea-induced denaturation and is able to rescue a mutant that is not sensitive to DGJ. It acts *in vivo* in synergy with galactose and with very low concentrations of DGJ.

## Methods and Materials

### Virtual screening

We selected a random subset of 9 million molecules (including FDA-approved drugs) from the ZINC database [23] for performing the virtual screening calculations in this study. Molecular docking calculations were carried out on the structure deposited in the PDB with the code 3S5Z [27] with the Leadfinder [28] docking program using default parameters. Protein structure 3S5Z contains 2 ligands: alpha-D-galactose (GLA) bound at the active site through D92, D93, K168, E203, R227, D231 (GLA site) and beta-D-galactose (GAL) bound at a different site through D255 and K374 (GAL site). Thus, two boxes (x, y, z dimensions 40 Å), centered on the position occupied by either GLA or GAL, were set for docking. Using the global shape similarity tool WEGA [29] alpha-D-galactose (the natural substrate of AGAL) was processed against the selected compound library and those molecules with the highest values for similarity score, ranging from 0.8 to 0.95, were selected for posterior docking studies. In total ten thousand purchasable molecules obtained after filtering with WEGA were selected for docking.

### Thermal unfolding

Thermal shift assay [30, 31] was adapted as described [32] using commercially available wt-AGAL, Fabrazyme^®^ (Genzyme, Cambridge, MA). Melting profiles were recorded under different conditions by thermal shift assay with the StepOne Real-Time PCR System (Applied Biosystems). The protein (0.5–1 mg/mL final concentration) was equilibrated in Na-Hepes 20 mM, NaCl 150 mM, pH 7.4 with Sypro Orange 2.5X (Invitrogen Molecular Probes, lifetechnologies.com), either without ligands or in the presence of ligands (6 mM DTP, 0.040 mM DGJ (SIGMA, Milan, Italy). The samples were distributed in 48-well PCR plates (0.025 mL in each well), sealed and heated from 20 to 90° at 1°C/min with increments of 0.6°C. The excitation wavelength of 490 nm and the emission wavelength of 575 nm, which are optimal for fluorescein, were adapted to detect Sypro Orange. Melting profiles of recombinant human phosphomannomutase2 were recorded similarly, the only exception was the presence of 1mM MgCl2 in the buffer. Phosphomannomutase 2 had been expressed and purified as described [33].

In order to verify the reversibility of the effect of DTP on Fabrazyme^®^, the enzyme equilibrated in Na-Hepes 20 mM, NaCl 150 mM, pH 7.4 was incubated for 1 hour at 4°C in the presence of 6 mM DTP dissolved in DMSO, then the sample was dialysed by ultrafiltration (by using Centrifugal ultrafiltration unit 15-mL MWCO 10 kDa, Merck-Millipore). A control experiment was also conducted incubating the enzyme with DMSO and dialysing it. Each dialysed sample was analysed by enzymatic assay (at pH 7.5) and by TSA (the experiment was conducted either in the presence of 6 mM DTP or with only DMSO) as described above.

### Urea-induced unfolding

The experiment was carried out with a method firstly described by Kim et al [34] and adapted as described [35]. In brief, Fabrazyme^®^ (0.32 mg/mL) was induced to unfold by urea in Hepes buffer at pH 7.4, with or without DTP 6 mM, in a final volume of 0.028 mL. The enzyme was incubated in the presence of urea concentrations ranging from 0 to 5 M. The samples were incubated at 10°C overnight, then each sample was treated with the appropriate amount of thermolysin necessary to obtain a 1:5 protease to protein ratio in the presence of CaCl_2_ (10 mM final concentration). After 1 min incubation at 37°C the reaction was stopped by the addition of EDTA (40 mM final concentration). The samples were separated by SDS-PAGE and coloured by Coomassie Blue Staining, then undigested proteins (Fabrazyme^®^ and thermolysin) were quantified with a ChemiDoc XRS (Bio-Rad Laboratories, Hercules, CA-USA). Two rectangular boxes were used to define specific protein bands, one for Fabrazyme^®^ and one for thermolysin as shown in S1 File. The intensity of bands was corrected subtracting the appropriate background (adjusted volumes), the ratio between the corrected values was calculated for Fabrazyme^®^ and thermolysin in each lane and was plotted against urea concentration.

### Transfection into COS-7 cells

COS-7cells were cultured in DMEM containing 10% FBS at 37°C and 5% CO2. The cells were transfected with individual plasmids containing mutant AGAL-encoding ORF using the LipofectAMINE2000 cationic lipid reagent in suspension [36].

67.5 micrograms of plasmid DNA in 13.5 mL Opti-MEM (Invitrogen) were mixed with 0.135 mL LipofectAMINE2000 reagent (Invitrogen) and incubated for 30 min at room temperature. COS-7 cells were harvested by trypsin treatment and resuspended in DMEM containing 10% FBS.

COS-7 in suspension (40.5 mL) were added to the transfection mix solution, distributed into 27 wells of six-well plates at 60% confluency and allowed to adhere for 5h.

The medium was substituted by fresh DMEM, 10% FBS, 1% DMSO (used to solubilize DTP and ambroxol) (1 mL), and drugs were added either in monotherapy or in binary combination as indicated in the specific figure legends. After 48 h incubation, the cells were washed in PBS (5 times), scraped and harvested by centrifugation. Dry pellets were resuspended in 0.030 mL of water and lysed by freeze-thawing. Three independent transfections were carried out for A230T, C56Y, C63Y, two for the other mutants, E341D, A37T, P40S, M42T, M42V, S126C, C172G, R118C, Q280K, L300F, L310F and G360C. Water-soluble extracts were used for enzyme assays or western blot. The Bradford colorimetric assay was used for protein quantification [37] using the Bio-Rad Protein Assay with bovine serum albumin as standard.

### Alpha-galactosidase assay

Tests on transfected mutants were carried out as described in [36]. Briefly: cell lysates (1–2 microliters) were added to 38 microliters of AGAL assay buffer (sodium citrate 27 mM-sodium phosphate dibasic 46 mM, 4-methylumbelliferyl-alpha-D-galactopyranoside 5 mM and N-acetyl-D-galactosamine 100 mM, pH 4.5) and incubated for 0.5–1 h at 37°C. All chemicals were obtained from Sigma. The reaction was stopped by adding 0.360 mL of 1 M sodium carbonate buffer [10]. Fluorescence was detected using a fluorescence spectrophotometer (Cary Eclypse-Varian) at 355 nm excitation and 460 nm emission.

A 4-methylumbelliferone standard curve ranging from 5 nM to 0.025 mM was run in parallel for conversion of fluorescence data to AGAL activity expressed as nmol/mg protein per min.

In order to measure the enzymatic activity in the presence of DTP 6 mM a spectrophotometric assay was run. Fabrazyme (1–2 microliters 0.25 mg/ml) were added to 0.038 mL of AGAL assay buffer (4-Nitrophenyl α-D-galactopyranoside 17 mM in Hepes 40 mM pH 7.4) and incubated for 3–15 min at 37°C. The reaction was stopped by adding 0.360 mL of 1 M sodium carbonate and the absorbance at 405nm was measured. The assay was conducted in the presence of DTP 6 mM (previously dissolved in DMSO) or DMSO.

### Western blot analysis

Western blot analysis for the detection of AGAL was performed using rabbit polyclonal antibodies (Abcam 70520) and HRP-coniugated anti-rabbit IgG antibody produced in goat (Bio-Rad 1706515). After SDS-PAGE (15% acrylamide), proteins were transferred to a PVDF membrane. The membrane was blocked with 5% (w/v) non-fat dried skimmed milk in blot solution at 4°C overnight, then treated with the primary antibody diluted in a blot solution 1:500 for 1 hour at room temperature. After washing, the detection was performed by using the Precision Plus Protein^™^ (Bio-Rad). GAPDH was revealed with mouse monoclonal anti lapine GAPDH (AbD seroTec (cat:4699–9555) 1:2000. All other chemicals were from Bio-Rad. Uncropped images of the gels are provided as S1 File.

### Deglycosylation

Deglycosylation of A230T expressed in COS7 cells (treated or untreated with DTP 6 mM) (10 μg f each) was performed according to the producer's instructions. wt-AGAL was processed at the same way as a control. Briefly, EDTA and SDS were added to the proteins (final concentrations were 20 mM and 0.1% respectively). The samples were then boiled for 5 min, immediately cooled before the addition of NP-40 to a final condition of 0.7% and N-Glycosidase F (0.5 unit) and incubated overnight at 37°C. A parallel experiment was conducted avoiding the denaturation step (SDS and heating) before adding NP-40. The samples was analysed by western blotting as described elsewhere.

### Bioinformatic and Statistical analysis

Standard deviations and p values (paired two-tailed Student's t-test) were obtained using Microsoft Excel (Microsoft Office professional 2010).

The effect of mutation on protein stability was predicted either running SDM on-line [38] or running MUPRO1.1[39] locally. Secondary structure was assigned with SEGNO [40]. Accessibility to solvent was calculated by SDM. Active site residues were identified with DrosteP [41]. Molecular dynamics was run with Yasara program under default conditions for 50 nsec [42] and flexibility assessed as described [43]. Protein damaging scores based on sequence conservation were derived using PolyPhen-2 software [44] or Fabry_CEP [45].

## Results

**Fig 1:**
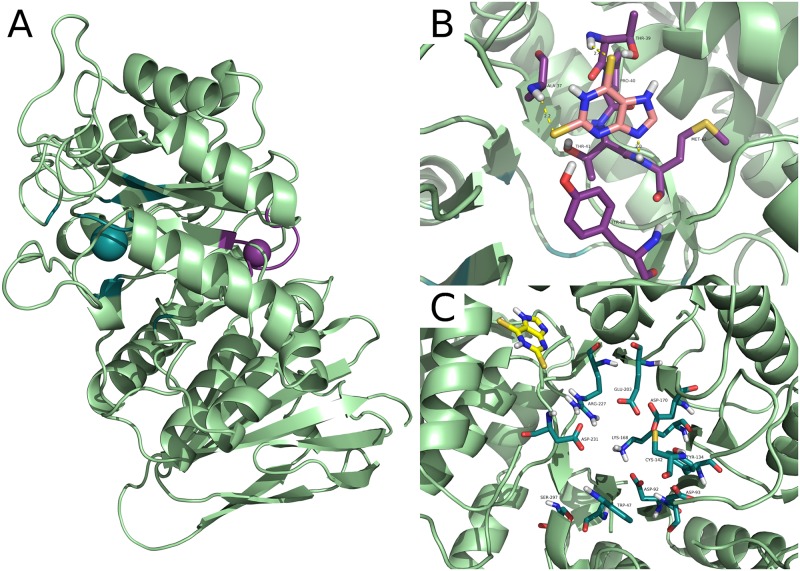
The active site and the allosteric binding site in human lysosomal alpha-galactosidase. The structure of lysosomal alpha-galactosidase is shown as a cartoon. In Panel A the active site (GLA site) is pinpointed by a blue sphere while the allosteric binding site is pinpointed by a violet sphere. Residues involved in the 2,6-dithiopurine allosteric and alpha-galactose binding site respectively are shown as sticks in Panel B or C respectively along with docked DTP. Predicted hydrogen bonds between DTP and AGAL within the allosteric binding site are shown as yellow dashed lines in Panel B. Best ranked pose of DTP in the alpha-galactose binding site area is shown in Panel C.

Molecular docking calculations were carried out on the AGAL structure deposited in the pdb with the code 3S5Z [27]. In this model AGAL binds two ligands per chain: alpha-D-galactose at the active site through D92, D93, K168, E203, R227 and D231 (GLA site) and beta-D-galactose at a different site through D255 and K374 (GAL site). This structure offers a good target to identify molecules that have little inhibitory activity and/or act in synergy with drugs directed against the active site. In proximity to the GAL site, we identified a hot-spot for binding that will be referred to as the allosteric site. It comprises residues A37, R38, T39, P40, T41, M42, E87, Y88 and does not overlap with the active site that is lined by W47, D92, D93, Y134, C142, K168, D170, E203, L206, Y207, R227, D231, S297 (Fig 1). The allosteric site is different from the GAL binding site.

We looked for molecules that bind the allosteric site with a high score, but bind the active site with a very low score. Drug repositioning, which should always be considered as the first choice when developing new therapies for rare diseases [46], could not be used because none of the approved drugs tested (FDA database) bound specifically the allosteric site (results not shown).

**Fig 2:**
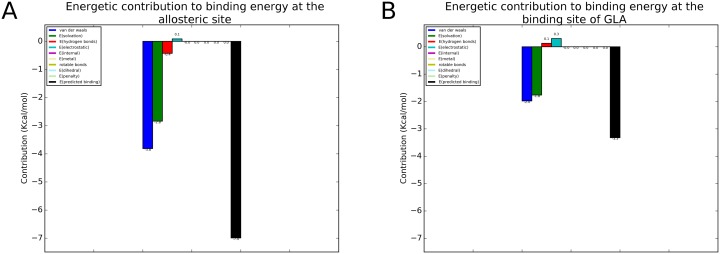
Energetic contributions to the binding of 2–6 dithiopurine to the allosteric site and the active site of human lysosomal alpha-galctosidase. Depicted energetic contributions are: van der Waals interactions (dark blue), volume-based free energy of solvation (green), hydrogen bonds (red), electrostatic energy (cyan), internal energy (pink), metal interactions (light yellow), energy of entropic losses associated with ligand’s rotatable bonds (dark yellow), dihedral energy (light blue), internal energy penalty (light green) and total predicted binding affinity (black). A) Decomposition for the allosteric site, and B), decomposition for the active site.

As a second choice we selected DTP, which is not an approved drug, but is a good candidate because it is safe when tested on human skin cells [25] and on mice [24] [26]. Even if the docking simulations started at GAL site DTP contacted the allosteric site. As a control we carried out a simulation limiting the search to a box centred on the active site as described in the methods. We show the interactions of DTP with the allosteric site in Fig 1B, and the corresponding energetic contributions in Fig 2A, the potential interactions of DTP with the active site (GLA site) in Fig 1C and the corresponding energetic contributions in Fig 2B. Docking simulations calculated a binding affinity at GLA’s binding site 52% lower (absolute value) than at the allosteric site (Fig 2). Scores calculated with Leadfinder [28] are reported as kcal/mol in Fig 2, but are meant only for rank-ordering different docking poses of DTP and cannot provide accurate binding energy predictions.

**Fig 3:**
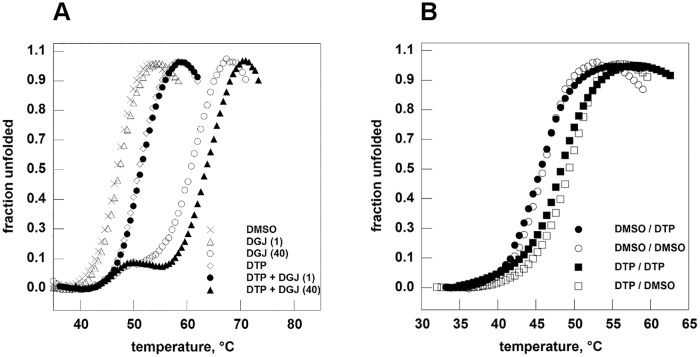
2–6 dithiopurine stabilizes human lysosomal alpha-galactosidase in thermal shift assay. Panel A. Fabrazyme^®^ (in Na-Hepes 20 mM, NaCl 150 mM, pH 7.4) was equilibrated in the presence of ligands dissolved in DMSO 20%: DGJ 1 microM (empty triangle) or 40 microM (empty circle); DTP 6 mM (empty diamond); DTP 6 mM plus DGJ 1 microM (filled circle); DTP 6 mM plus DGJ 40 microM (filled triangle). A control (with only DMSO) was also shown (x). Panel B. Fabrazyme^®^ (in Na-Hepes 20 mM, NaCl 150 mM, pH 7.4) was incubated in the presence of DTP 6 mM for 1 hour at 4°C then DTP was eliminated by dialysis. A control experiment was conducted by incubating the enzyme only with DMSO. Both the aliquots of Fabrazyme^®^ were analysed by thermal shift assay in the presence of DTP 6 mM or in the presence of DMSO. Filled squares: DTP/DTP; open squares: DMSO/DTP; filled circles: DTP/DMSO; open circles: DMSO/DMSO where the first word of the label corresponds to the pretreatment and the second part corresponds to the presence of the compound during the thermal scan. The protein samples were heated from 20 to 90° at 1°C/min in the presence of Sypro Orange 2.5x. Data were shown as normalized curves.

DTP does not inhibit AGAL (Fabrazyme^®^, Genzyme) when tested at 6 mM concentration using 4-Nitrophenyl α-D-galactopyranoside 17 mM in Hepes 40 mM pH 7.4, whereas DGJ blocks completely the enzyme (1% residual activity) at concentration as low as 0.001 mM.Thermal shift assay was used to evaluate the ability of DTP (6mM) to stabilize wt-AGAL, (Fabrazyme^®^, Genzyme) in the presence or in the absence of DGJ. DTP is able to stabilize the enzyme against thermal denaturation alone or in synergy with the iminosugar (Fig 3A). without forming covalent bonds. To prove the reversibility of this stabilising effect, DTP (6 mM) was incubated with enzyme and subsequently removed by dialysis before thermal shift assay. Results are indicated in Fig 3B (filled squares DTP/DTP; open squares DMSO/DTP; filled circles; DTP/DMSO; open circles DMSO/DMSO) where the first word of the label corresponds to the pretreatment and the second part corresponds to the presence of the compound during the thermal scan. The specificity of effect of DTP was tested running thermal shift assay on phosphomannomutase2, an enzyme structurally and functionally unrelated to AGAL in the presence of DTP 6 mM or a positive control, glucose 1–6 bisphosphate 0.5 mM (S1 Fig).

**Fig 4:**
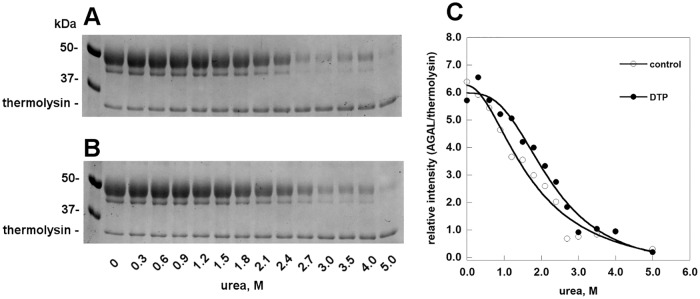
2–6 dithiopurine protects human alpha-galactosidase against urea-induced unfolding. Fabrazyme^®^ was incubated in the presence of urea (from 0 to 5 M), with (B) or without (A) DTP 6 mM, at 10°C over night, then each sample was treated for 1 min at 37°C with thermolysin (1:5 protease to protein ratio). Folded undigested proteins were visualized by SDS-PAGE. The intensity of the bands was quantified and the relative intensity of major band of Fabrazyme^®^ and thermolysin in each lane was plotted against urea concentration (C).

The stability of AGAL in the presence or in the absence of DTP was tested by unfolding induced by a range of urea concentrations, followed by limited proteolysis. The enzyme unfolds at 2.7 M urea in the absence of DTP, and at 3 M urea in the presence of DTP (Fig 4). A minor band, which might represent a differently glycosylated form of AGAL, is observed in Fig 4. Its intensity correlates with that of the major band (data not shown).

AGAL mutants were expressed transiently in COS-7 cells. We tested the effect of DTP on the mutant A230T [47], which is non-responsive to DGJ [36], but eligible for PC therapy [48].

**Fig 5:**
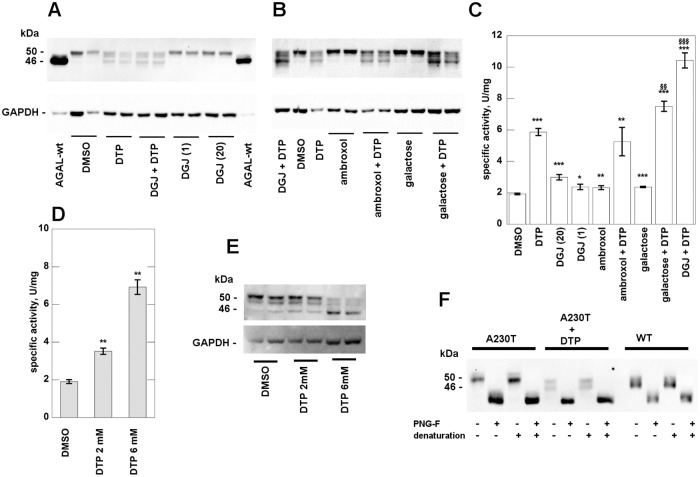
2–6 dithiopurine rescues mutant human alpha-galactosidase in monotherapy or in synergy. COS-7 cells were cultured in conventional medium and transfected with plasmid containing A230T mutant. Cells were treated with: DTP 6 mM, DGJ 1 microM, DGJ 20 microM, ambroxol 40 microM, ambroxol 40 microM plus DTP 6 mM, galactose 100 mM, galactose 100 mM plus DTP 6 mM, DGJ 1microM plus DTP 6 mM. All the molecules were dissolved in DMSO and an appropriate control was realized. After 48 h incubation, the cells were scraped and lysed then water-soluble extracts were analysed by western blotting (A and B) and enzyme assay (C). The effect of DTP 2 mM is shown in D and E. Cell extracts were treated with N-Glycosidase F and then analysed by WB (F). Standard deviations is indicated by bars and differences that are statistically significant are flagged by * when compared to the control with DMSO (p < 0.05:*; p < 0.01: **; p < 0.001:***), are flagged by § when compared to DTP (p < 0.01:^§§^; p < 0.001:^§§§^).

After transfection of A230T into COS-7 cells, specific bands were revealed by Western blot (WB) and the total alpha-galactosidase activity was measured in cell extracts. In order to interpret results it is worth remembering that AGAL is synthesized as a precursor of 50 kDa, but it is converted into a mature form of 46 kDa. A detailed analysis of Ishii and co-workers [49] demonstrated that when AGAL mutants are correctly processed into a 46 kDa form, they are also transferred into lysosomes. In Fig 5A we show the migration of transfected wt-AGAL (90±9 U/mg activity in the lysates) as a reference. A230T shows a faint band corresponding to the precursor form of AGAL. This is not induced by the treatment with acknowledged PCs, DGJ, galactose [50] or ambroxol [21], at the dosages commonly used to measure their chaperoning effect on transfected mutants, 0.020 mM, 100 mM or 0.040 mM respectively. However, DTP 6 mM promotes the maturation of A230T. Moreover, its effect is potentiated by galactose and DGJ, even at very low concentration (0.001mM), but not by ambroxol (Fig 5A, 5B and 5C).

The lowest concentration at which DTP promotes A230T maturation is 2 mM (Fig 5D and 5E). The different bands visualised on WB represent different glycosylated forms, as demonstrated by treatment of A230T or wt-AGAL with N-Glycosidase F Fig 5F.

**Fig 6:**
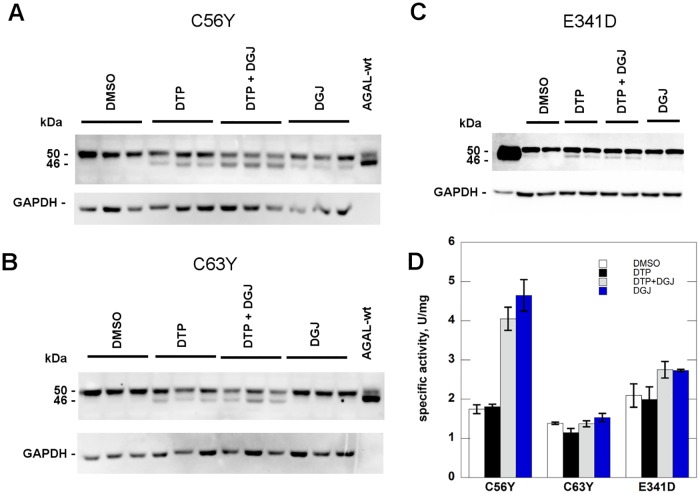
2–6 dithiopurine promotes processing of some mutant human alpha-galactosidase. COS-7 cells were cultured in conventional medium and transfected with plasmid containing C56Y, C63Y and E341D mutants. Cells were treated with: DTP 6 mM, DGJ 1 microM, DGJ 1 microM plus DTP 6 mM. All the molecules were dissolved in DMSO and an appropriate control was realized. After 48 h incubation, the cells were scraped and lysed then water-soluble extracts were analysed by western blotting (A, B and C) and enzyme assay (D). Standard deviations are indicated by bars.

We tested the effect of DTP 6 mM on further mutants expressed in COS-7 cells: C56Y (Fig 6A), C63Y (Fig 6B) and E341D (Fig 6C). In these cases, enzymatic activity was not recovered (Fig 6D), possibly because the proteins are not catalytically active, but increased protein processing was detectable. C63Y and E341D are not responsive do DGJ [12, 13]. C56Y activity increases in the presence of DGJ although it does not meet the criteria chosen Lukas *et al* [13] to define responsiveness.

**Fig 7:**
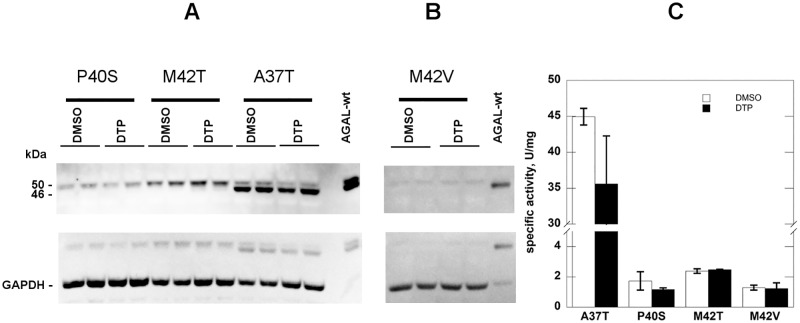
2–6 dithiopurine has no effect on mutations affecting the allosteric site. COS-7 cells were cultured in conventional medium and transfected with plasmid containing A37T, P40S, M42T, M42V mutants. Cells were treated with DTP 6 mM dissolved in DMSO and an appropriate control was realized. After 48 h incubation, the cells were scraped and lysed then water-soluble extracts were analysed by western blotting (A and B) and enzyme assay (C). Standard deviations are indicated by bars.

We tested four mutations affecting residues in the allosteric site, A37T, P40S, M42T, M42V. None of these mutants responds to DTP (Fig 7). A37T [12], M42T [13] and M42V [8, 9, 12] are responsive to 0.020 mM DGJ, whereas P40S is not [10, 11, 13]. This finding supports the idea that DTP binds the allosteric site found *in silico*, although it does not prove it, because the same molecule has no effect on mutations that do *not* affect the allosteric site. An example is provided by S126C, C172G, and R118C which are not responsive to DGJ, [10–12, 51] (S2 Fig) and by Q280K, L300F, L310F and G360C (S3 Fig), which are responsive to DGJ [12] [36].

## Discussion

The catalytic domain of AGAL is formed by a TIM-barrel and the active site is located at the carboxyterminal end of beta-strands, as expected [52].

We identified a different druggable pocket located at the opposite side of the TIM-barrel of AGAL. Some molecules resulting from our virtual screening bind better to the allosteric site than to the active site. One of these molecules, a chemopreventive 2,6-dithiopurine DTP, appeared particularly promising because it is actively transported into mammalian cells where it accumulates at millimolar concentration [25] and it is safe [24] [26].

DTP promotes the processing of some mutants of AGAL that are not responsive to DGJ and in one case, A230T, enhances its activity in monotherapy as well as in synergy with DGJ or galactose.

Not all missense mutations associated with FD are responsive to PC and we have not yet found molecular features that are necessary and sufficient to determine responsiveness. Mutations occurring at non conserved sites are generally less severe and responsive to PC, but there are exceptions to this rule. The best example we encountered is A230T, a mutation occurring at a non conserved site. A230 is exposed to solvent and exhibits moderate flexibility (Root mean square fluctuation, RMSF, of alpha carbon 0.74 ±0.02 Å). A230 is not directly implicated in catalysis, but it flanks D231, a proton donor that is essential for the hydrolysis of the substrate and belongs to a short stretch in polyproline II conformation. Its mutation to Threonine is classified as mildly destabilizing by MUPRO [39] or SDM [38]. The position specific substitution score, which measures the tolerability of the mutation in orthologous sequences and correlates with responsiveness to DGJ, is benign [48]. A230T is classified as possibly damaging or benign by HumDiv- and HumVar-trained PolyPhen-2 models respectively [44]. To summarize, A230T is eligible for PC therapy and does respond to DTP, although it does not respond to DGJ. Although DTP might be active only on a minority of AGAL missense mutations, we suggest that it should be tested in the cases that receive a positive score with Fabry_CEP [45], but fail to respond to DGJ. We are aware that the interaction of DTP with AGAL, which occurs in vitro only at high concentration, cannot exclude non specific off-target effects of the drug on protein homeostasis. Derivatives of DTP obtained by techniques of lead optimization and medicinal chemistry might prove to be effective at lower doses and more specific.

## Supporting Information

S1 Fig 2–6 dithiopurine does not stabilizes human lysosomal phosphomannomutase2 in thermal shift assay. Human Phosphomannomutase2 (in Na-Hepes 20 mM, NaCl 150 mM, MgCl_2_ 1mM pH 7.4) was equilibrated in the presence of ligands dissolved in DMSO 20%: DTP 6 mM (empty circle) and glucose 1, 6 bisphosphate (G16) 0.5 mM (filled squares) as a positive control. A control (with only DMSO) was also shown (filled circles).
(TIF)

S2 Fig 2–6 dithiopurine has no effect on some mutations that are not responsive to DGJ. COS-7 cells were cultured in conventional medium and transfected with plasmid containing R118C, S126C and C172G mutants. Cells were treated with: DTP 6 mM, DGJ 1 microM, DGJ 1 microM plus DTP 6 mM. All the molecules were dissolved in DMSO and an appropriate control was realized. After 48 h incubation, the cells were scraped and lysed then water-soluble extracts were analysed by western blotting (A, B and C) and enzyme assay (D). Standard deviations are indicated by bars.
(TIF)

S3 Fig 2–6 dithiopurine has no effect on some mutations that are responsive to DGJ. COS-7 cells were cultured in conventional medium and transfected with plasmid containing L310F, L300F, Q280K and G360C mutants. Cells were treated with DTP 6 mM. All the molecules were dissolved in DMSO and an appropriate control was realized. After 48 h incubation, the cells were scraped and lysed then water-soluble extracts were analysed by western blotting (A, B) and enzyme assay (C). Standard deviations are indicated by bars.
(TIF)

S1 File Uncropped gels. Images are relative to gels shown in Figs 4, 5, 6 and 7.
(PDF)

## Abbreviations

FD: Fabry disease
AGAL: lysosomal alpha-galactosidase
ERT: Enzyme replacement therapy
DGJ: 1-deoxygalactonojirimycin
DTP: 2,6-dithiopurine
GLA: alpha-D-galactose
GAL: beta-D-galactose
