# Supplementary figures and images for: Identification of an Allosteric Binding Site on Human Lysosomal Alpha-Galactosidase Opens the Way to New Pharmacological Chaperones for Fabry Disease
Source: PLoS One. 2016 Oct 27;11(10):e0165463. doi: 10.1371/journal.pone.0165463 (PMC5082870; doi:10.1371/journal.pone.0165463)

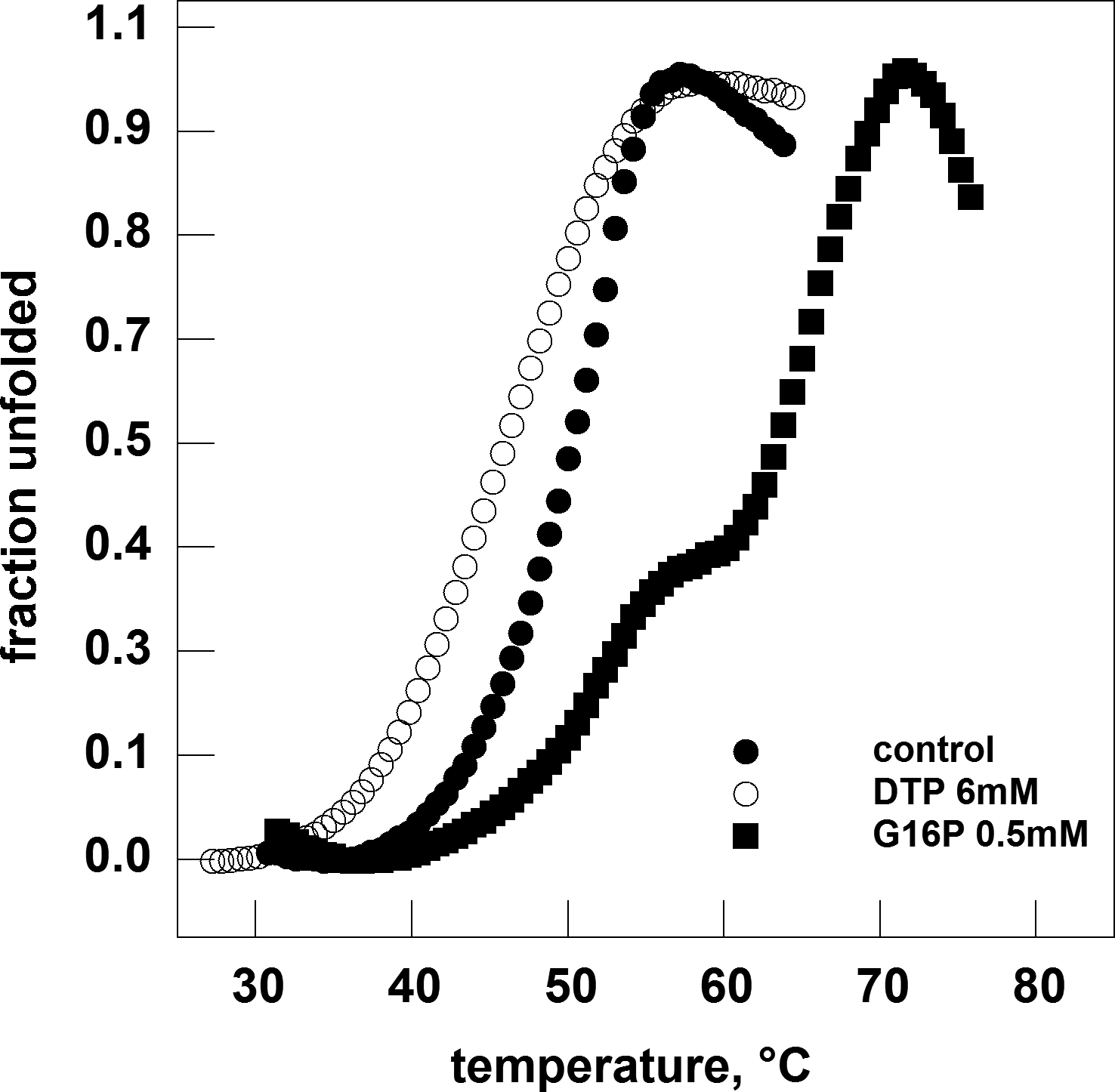

Supplement: S1 Fig — Human Phosphomannomutase2 (in Na-Hepes 20 mM, NaCl 150 mM, MgCl2 1mM pH 7.4) was equilibrated in the presence of ligands dissolved in DMSO 20%: DTP 6 mM (empty circle) and glucose 1, 6 bisphosphate (G16) 0.5 mM (filled squares) as a positive control. A control (with only DMSO) was also shown (filled circles). (TIF) [file pone.0165463.s001.tif]

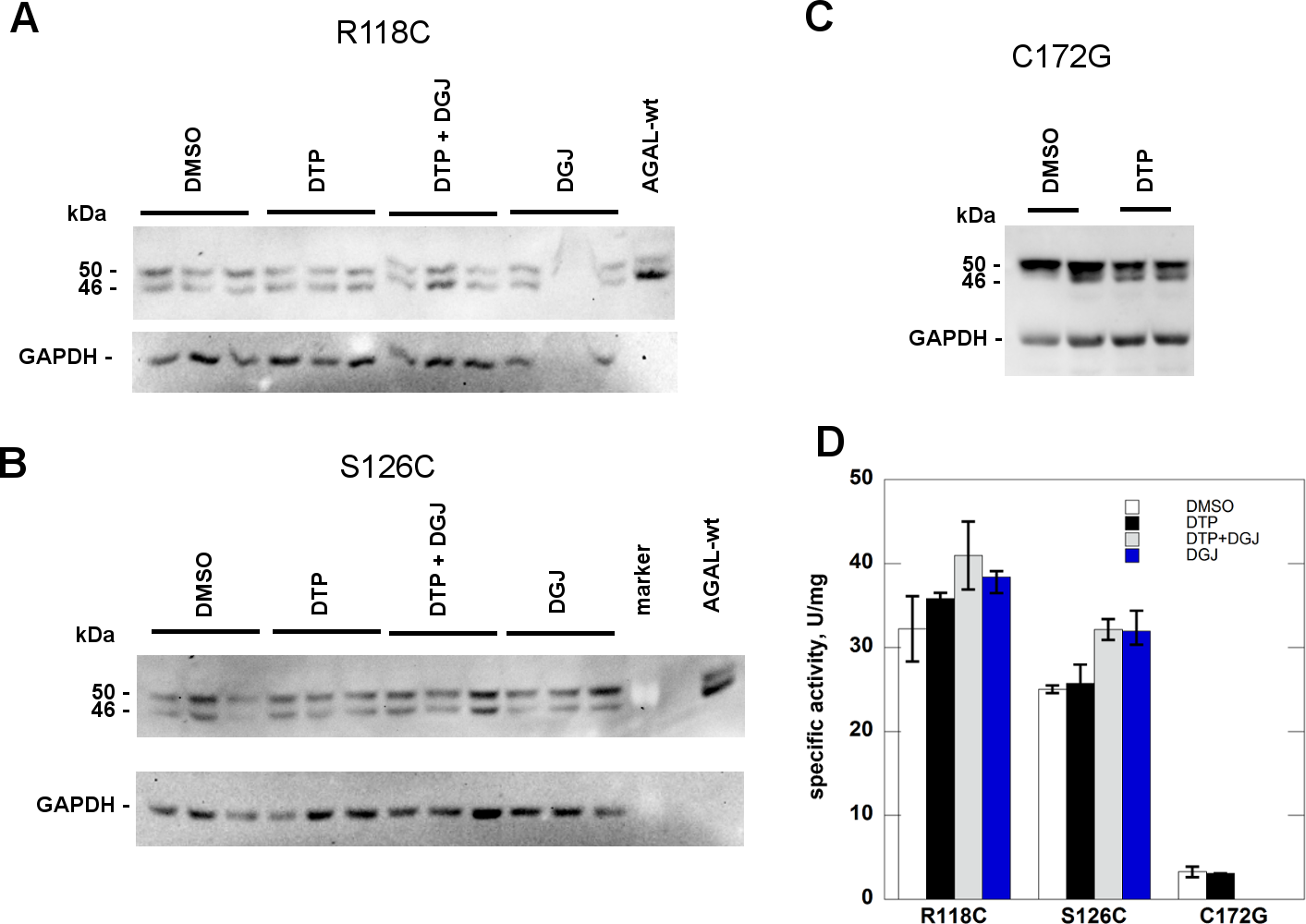

Supplement: S2 Fig — COS-7 cells were cultured in conventional medium and transfected with plasmid containing R118C, S126C and C172G mutants. Cells were treated with: DTP 6 mM, DGJ 1 microM, DGJ 1 microM plus DTP 6 mM. All the molecules were dissolved in DMSO and an appropriate control was realized. After 48 h incubation, the cells were scraped and lysed then water-soluble extracts were analysed by western blotting (A, B and C) and enzyme assay (D). Standard deviations are indicated by bars. (TIF) [file pone.0165463.s002.tif]

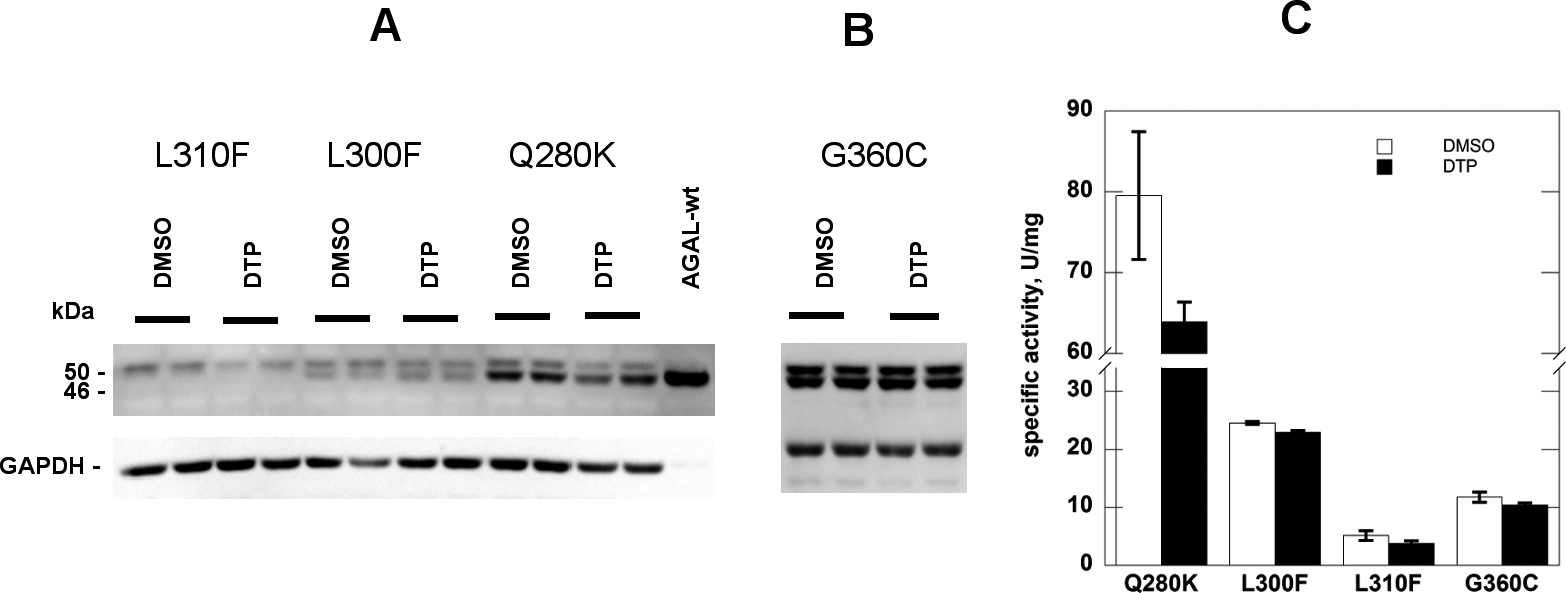

Supplement: S3 Fig — COS-7 cells were cultured in conventional medium and transfected with plasmid containing L310F, L300F, Q280K and G360C mutants. Cells were treated with DTP 6 mM. All the molecules were dissolved in DMSO and an appropriate control was realized. After 48 h incubation, the cells were scraped and lysed then water-soluble extracts were analysed by western blotting (A, B) and enzyme assay (C). Standard deviations are indicated by bars. (TIF) [file pone.0165463.s003.tif]
