# Supplementary material for: Identification of an Allosteric Binding Site on Human Lysosomal Alpha-Galactosidase Opens the Way to New Pharmacological Chaperones for Fabry Disease
Source: PLoS One. 2016 Oct 27;11(10):e0165463. doi: 10.1371/journal.pone.0165463 (PMC5082870; doi:10.1371/journal.pone.0165463)

Uncropped original from Fig 4 control

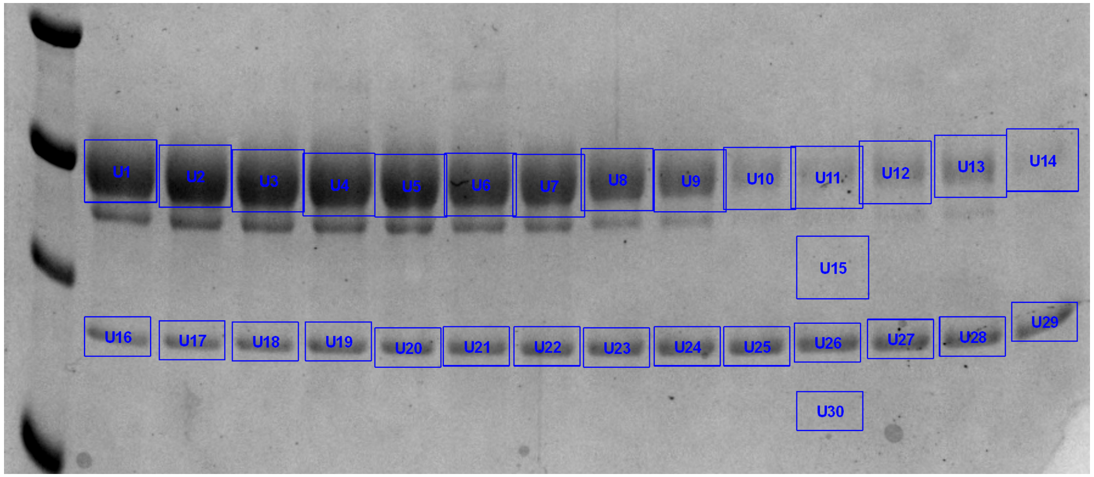

Uncropped original from Fig 4 +DTP

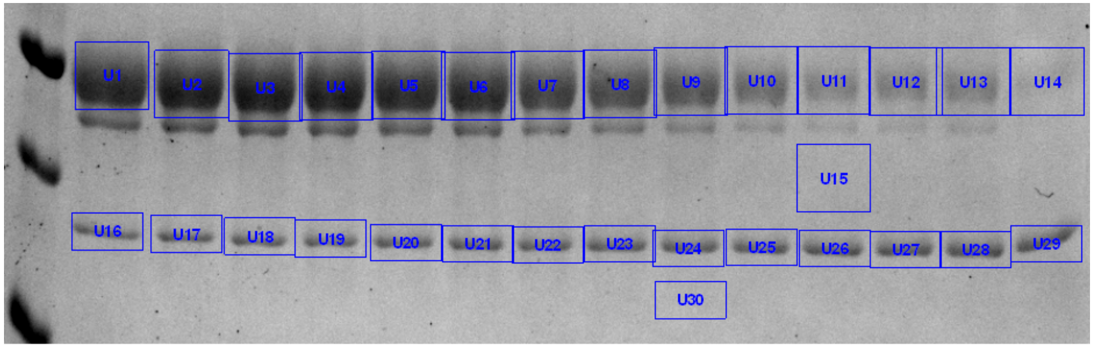

Uncropped original Western blots from Fig 8

Panel A

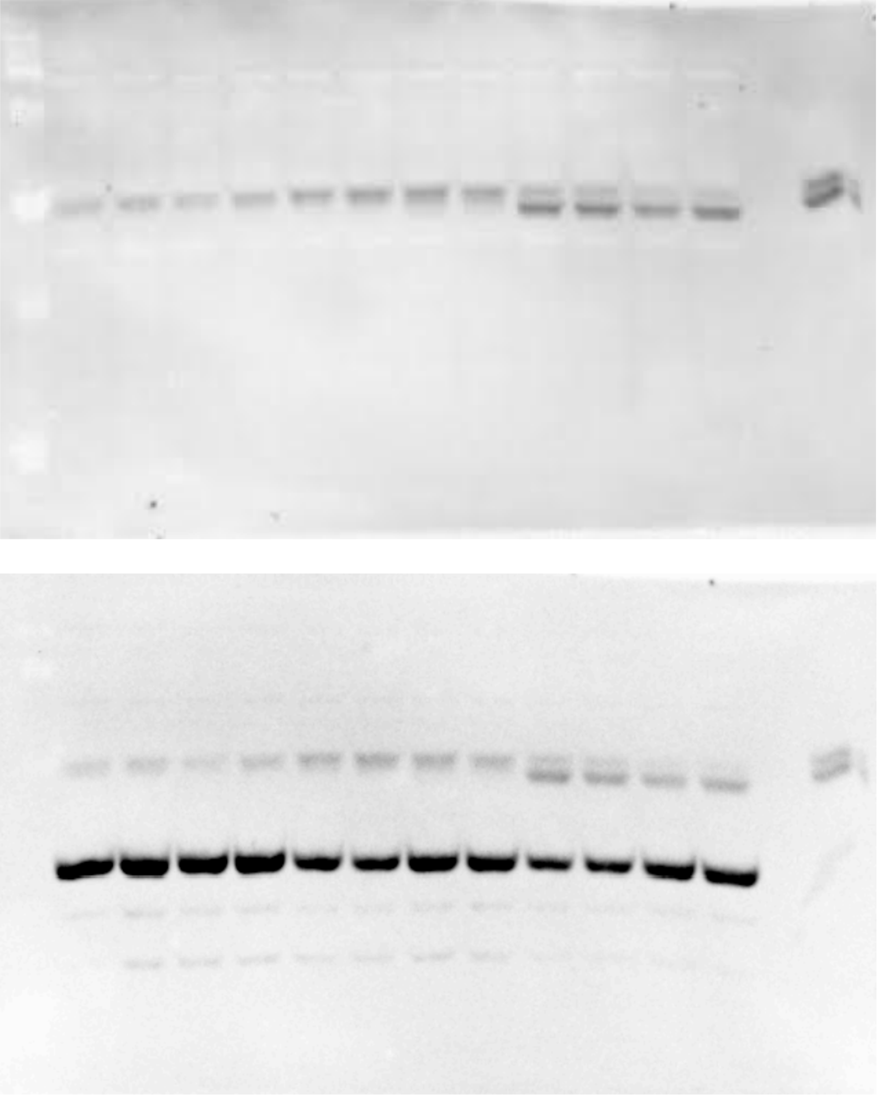

Panel B

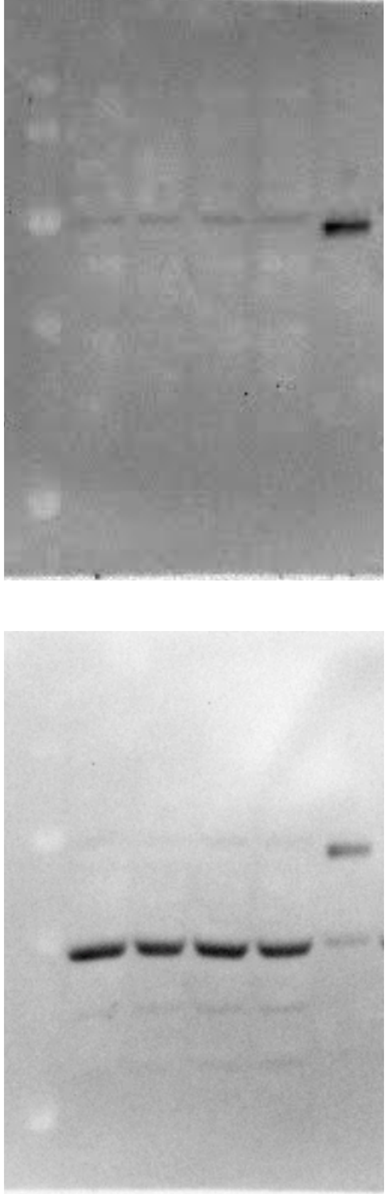

Uncropped original Western blots from Fig 7

Panel A

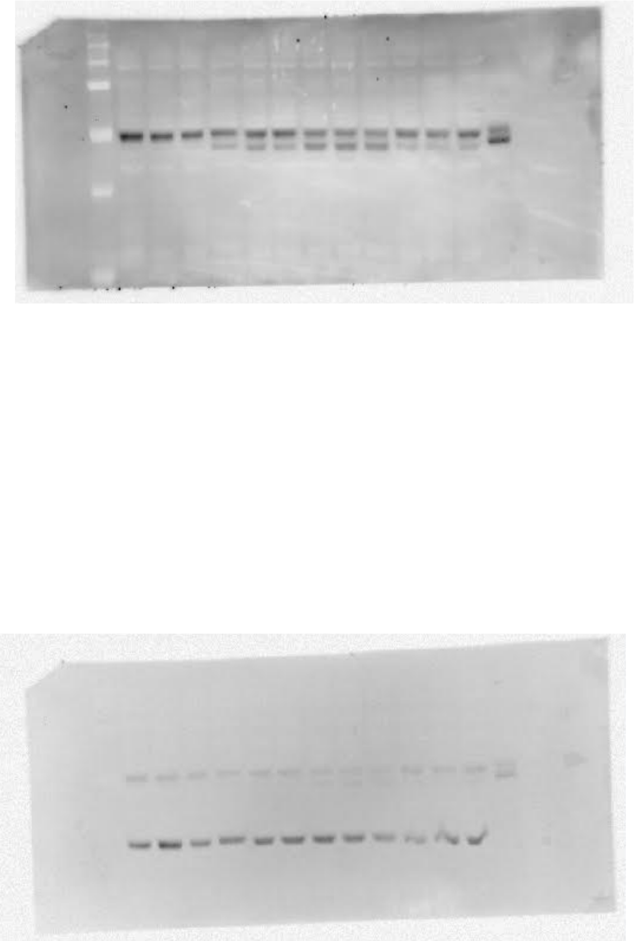

Panel B

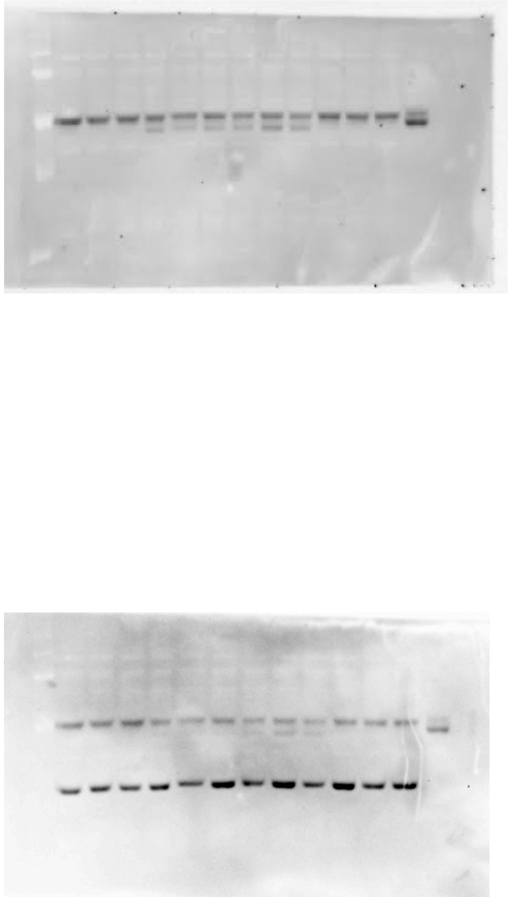

Panel C

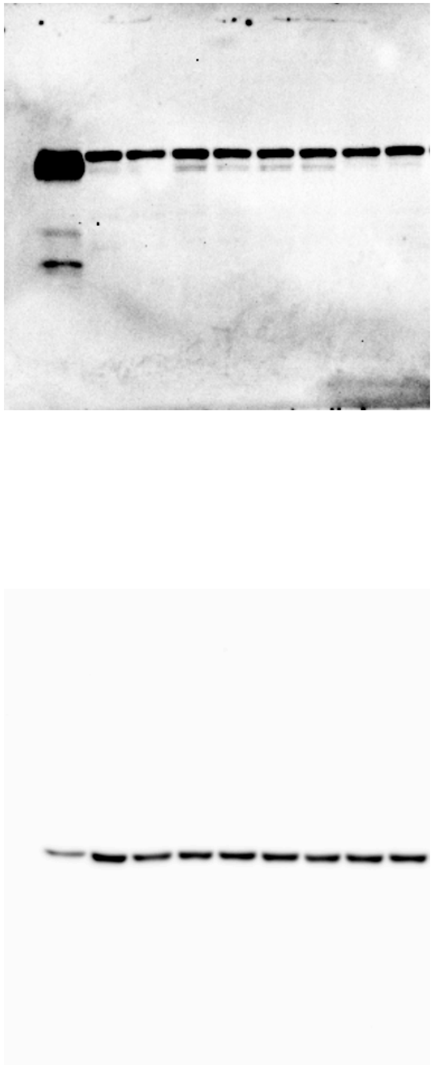

Uncropped original Western blots from Fig 6

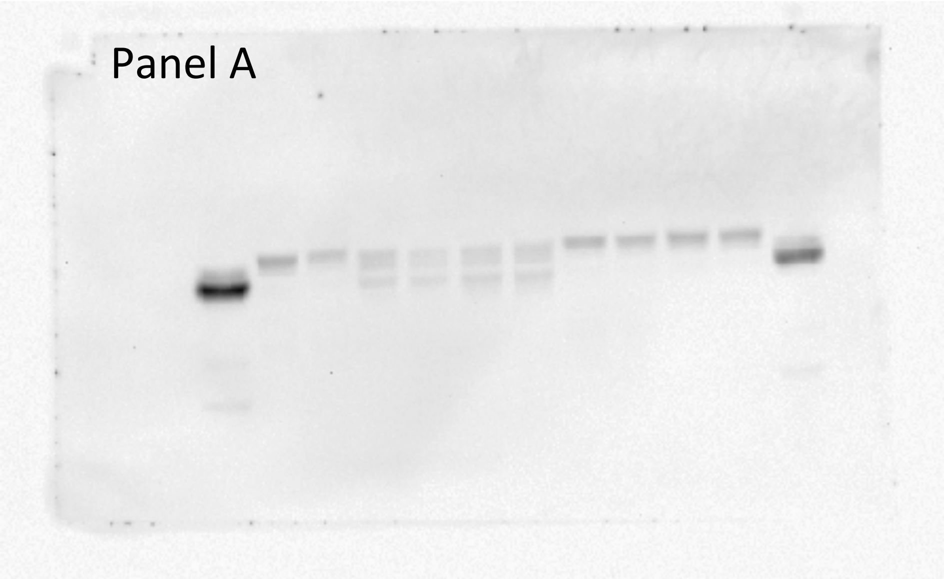

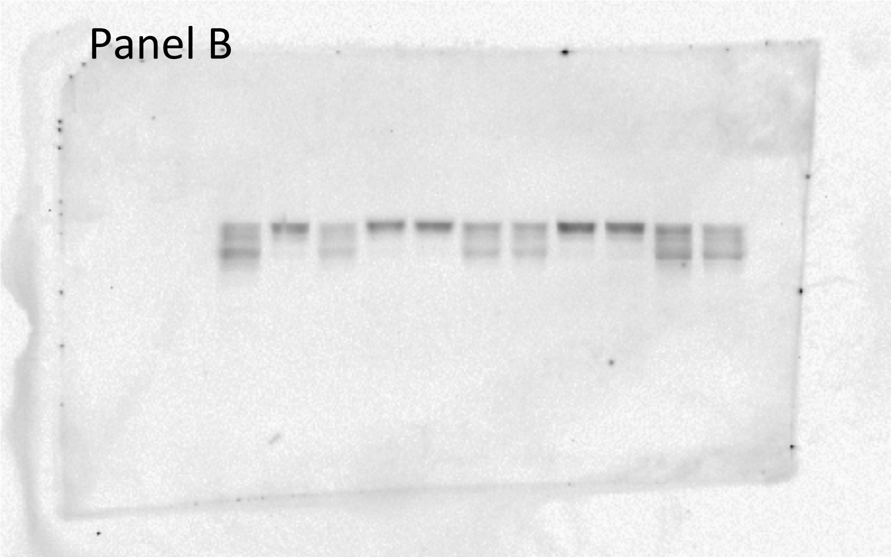

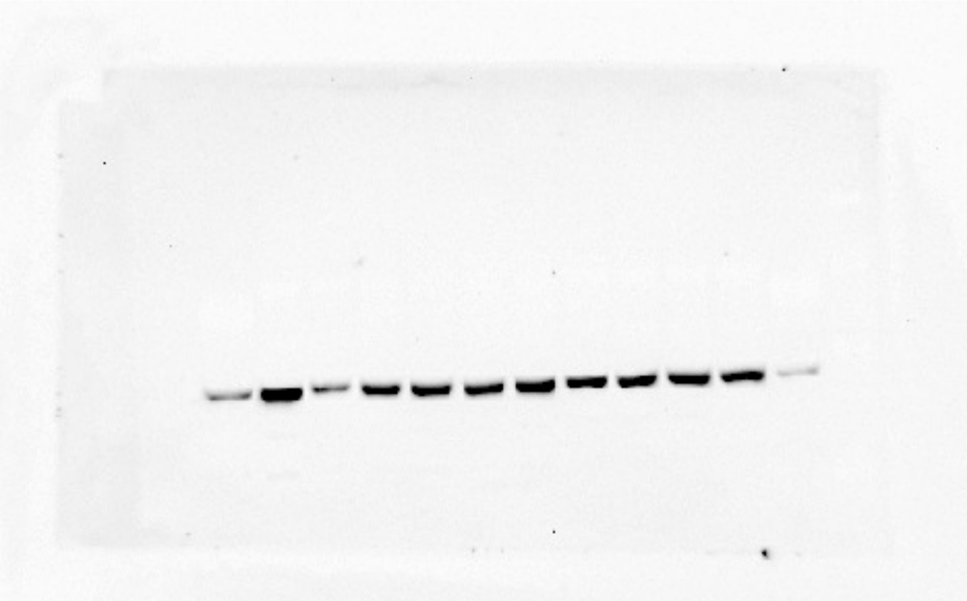

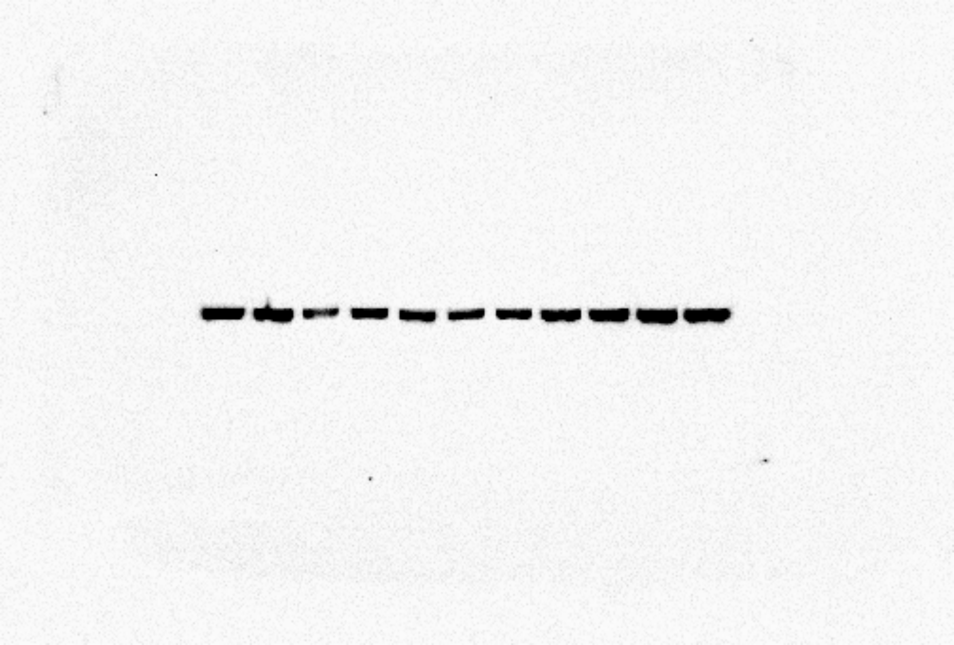

Uncropped original Western blots from Fig 6

Panel E

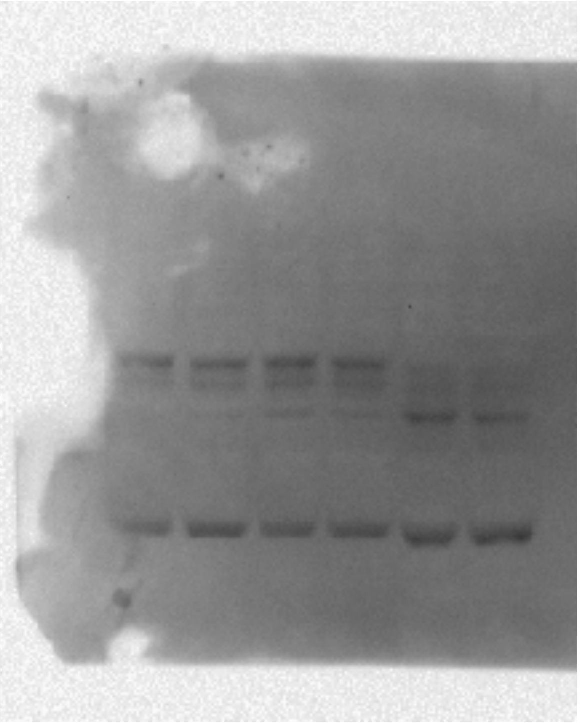

Panel F

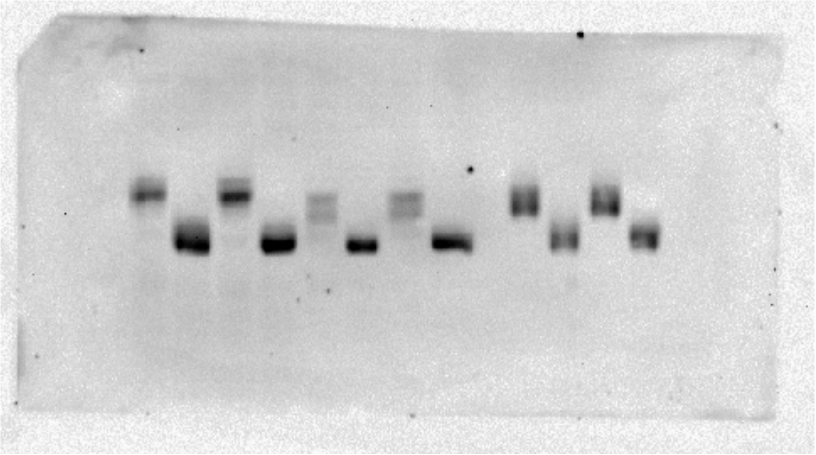

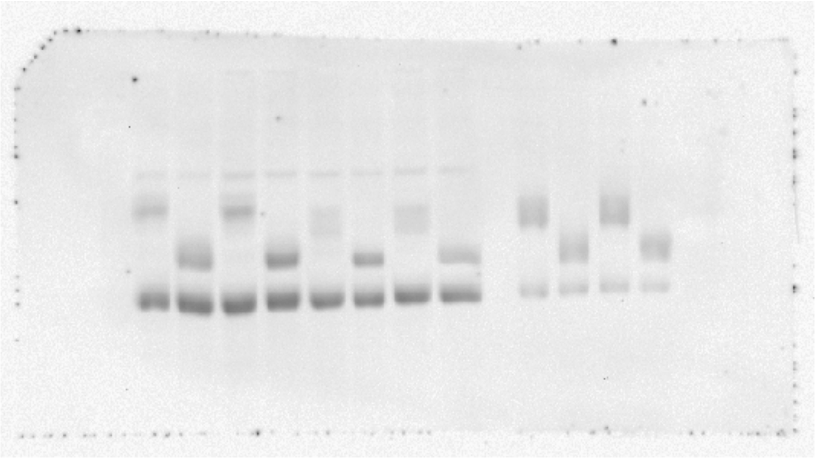

Supplement: S1 File — Images are relative to gels shown in Figs 4, 5, 6 and 7. (PDF) [file pone.0165463.s004.pdf]
